# Aerosolization Affects *Bacillus globigii* Vegetative Cell and Spore Behaviors

**DOI:** 10.3390/microorganisms13112532

**Published:** 2025-11-05

**Authors:** Brooke L. Smith, Meiyi Zhang, Sunil Kumar, Maria D. King

**Affiliations:** Department of Biological and Agricultural Engineering, Texas A&M University, College Station, TX 77843, USA; blsmith5@tamu.edu (B.L.S.); teresa1217@tamu.edu (M.Z.); sunil.kumar@ag.tamu.edu (S.K.)

**Keywords:** bacterial aerosol, *Bacillus globigii*, aerosolization, environmental effects, antimicrobial resistance, wetted wall cyclone

## Abstract

Antimicrobial resistance (AMR) in bacteria is a critical global health threat, yet the impact of environmental stressors such as aerosolization on resistance remains unclear. We previously showed that aerosolization can induce antibiotic resistance in *Escherichia coli* MG1655, a gram-negative pathogen simulant. Here, we investigated *Bacillus globigii*, a surrogate for the gram-positive pathogen *Bacillus anthracis*, to assess how aerosolization affects bacterial survival and antibiotic resistance. *B. globigii* vegetative cells and spores were aerosolized under varying conditions and durations (5, 10, 15, 30, and 45 min) into a sterile, airtight chamber and collected using the wetted wall cyclone (WWC) system. Samples were analyzed via antibiotic susceptibility testing, culture-based assay, and quantitative polymerase chain reaction (qPCR). Vegetative cells exhibited the lowest culturability after 5 and 30 min aerosolization, while spores showed reduced culturability at 15–45 min. Both vegetative cells and spores displayed lowest antibiotic susceptibility profiles after 15 min of aerosolization. Our findings suggest that aerosolization duration and bacterial state (vegetative vs. spores) can influence bacterial survival and development of antibiotic resistance. Understanding these dynamics is essential for designing strategies to mitigate the airborne spread of antibiotic-resistant bacteria.

## 1. Introduction

Antimicrobial resistance (AMR) continues to threaten global health. It contributed to almost 5 million deaths globally in 2019 and continues to limit effective treatment options for bacterial infections [1,2]. Not only does AMR pose a significant threat to public health, it also creates economic burdens to society, which are predicted to result in trillions of dollars’ worth of additional healthcare costs by 2050 and gross domestic product (GDP) losses annually by 2030 [3]. While the overuse of antibiotics in therapeutics and agriculture is a well-recognized driver, environmental stressors are increasingly being studied as additional contributors to resistance evolution and dissemination, due to the frequent detection of drug-resistant organisms in environments other than hospitals [4,5,6,7,8,9]. Airborne transmission of microbes with AMR is of particular concern because bioaerosols can facilitate the spread of resistant bacteria across long distances and between rooms [10,11,12]. A study of livestock farm aerosols revealed a significant positive association between AMR in *Staphylococcus* spp. for common antibiotics and farm-level antimicrobial usage, regardless of sampling seasons and livestock groups [13]. Our previous studies have demonstrated that aerosolization can trigger stress-induced resistance responses—an immediate response involving membrane efflux pumps after short aerosolization periods and a second mechanism of lipid biosynthesis upregulation after longer aerosolization periods—in *Escherichia coli* MG 1655, a gram-negative surrogate strain for pathogenic bacteria [14,15,16]. These findings highlight how environmental stressors, such as mechanical shear, osmotic shock, and desiccation, may accelerate bacterial adaptation in ways not captured by traditional antibiotic exposure studies [17,18,19,20]. However, little is known about whether gram-positive bacteria—especially spore-forming organisms—exhibit similar behaviors under aerosolization stress.

*Bacillus globigii* (also known as *Bacillus atrophaeus*) is a nonpathogenic surrogate for the gram-positive pathogen *Bacillus anthracis*, the causative agent of anthrax, due to their similarities in size and shape, spore-forming ability, and its distinctive orange pigmentation enabling easier enumeration and differentiation from other environmental microbes [21,22,23]. The dual life cycle of vegetative cells and dormant spores makes *B. globigii* an ideal model for understanding stress responses across different physiological states. Vegetative cells are metabolically active but susceptible to stress, whereas spores are metabolically dormant, highly resistant to physical and chemical antimicrobials, and capable of long-term survival [24,25]. *B. globigii* spores are surrounded by a proteinaceous coat, an outer membrane, a peptidoglycan cortex, and an inner membrane, with a central core containing DNA, ribosomes, and enzymes [22,26]. In the sporulation process, the dividing cell forms an asymmetrical septum, creating a smaller forespore with its own DNA and the introversion of the cell’s inner membrane, which is then engulfed by the larger cell, resulting in a double membrane structure surrounding the forespore. After introversion, the proteinaceous layer and peptidoglycan cortex are synthesized, followed by the formation of the outer coat of proteins and spore maturation [27,28].

Gram-positive bacteria are a diverse subset of bacteria that can survive in many different environments. Gram-positive bacteria have a rapid response to stressors including physical responses from an inner membrane and a thick cell wall. These cells also respond to stressors genetically, for example, by using the sigma factor, which is a gene system that helps the cells respond to various environmental stressors [29,30]. Gram-positive bacteria are resilient and can cope with a variety of environmental conditions. Adaptability is what allows the resilience of gram-positive bacteria. Studies have shown that *Bacillus* spores are more resistant to extreme stresses, including desiccation, solvents, osmolarity, pH, ultraviolet light, and γ-radiation, yet the impact of aerosolization on their antibiotic susceptibility remains underdefined [30,31]. Addressing this knowledge gap is critical for understanding the potential for resistant gram-positive pathogens to disseminate through airborne routes.

Bacteria encounter various stressors—changes in relative humidity (RH), temperature, osmotic changes, mechanical stressors, and air contaminants—when entrained in the air. As opposed to *E. coli* cells, gram-positive bacteria can withstand a higher turgor pressure of approximately 20–25 atm [32,33]. In this study, we aerosolized *B. globigii* under controlled conditions and collected them using a wetted wall cyclone (WWC) and a high-velocity, low cutpoint WWC (LCP-WWC) to investigate the culturability, genomic integrity, and antibiotic susceptibility profile of both vegetative cells and spores [34]. By comparing stress responses across different physiological states and sampling methods, this study provides new insights into how aerosolization influences AMR in gram-positive bacteria. Although bacterial spores are metabolically dormant in their desiccated states, they can become transiently vulnerable to environmental stress during resuspension and early germination, when rehydration of the spore core and cortex reduces resistance properties and increases permeability to external stressors [35]. Based on this understanding, we hypothesized that the culturability of vegetative cells would be higher than that of spores across different aerosolization times and between the two collection systems. However, the degree of change in antibiotic susceptibility, reflected by the inhibition zone diameter to commonly tested antibiotics, would be greater for spores, particularly under high-velocity collection by the LCP-WWC and when archived for 15 days.

## 2. Materials and Methods

### 2.1. Aerosolization Chamber and Experimental Setup

Experiments were conducted in a custom-built sealed and insulated aerosol chamber (27 L, polypropylene/polyethylene) as described previously [14,15]. Aerosols were generated using a six-jet Collison nebulizer (BGI, Waltham, MA, USA) operated at 138 kPa (20 psi), which produces an output of 12 L/min. The nebulizer was connected to the chamber via sterile 1″ vinyl tubing. A WWC and an LCP-WWC were used to collect bioaerosols directly into sterile Milli-Q water. An internal propeller maintained air circulation at 80 L/min volumetric flow rate, approximating 0.7 m/s linear velocity in a ventilated room. Temperature and RH were continuously logged using a HOBO data logger (HOBO Data Loggers, Bourne, MA, USA). Prior to each experiment, all components were autoclaved or disinfected with 5% bleach and 70% isopropanol following previously established protocol [36]. Chamber decontamination and HEPA-filtered air supply were used to minimize contamination.

### 2.2. Preparation of Bacillus globigii Cultures

*B. globigii* vegetative cells were prepared by streaking from frozen stock onto tryptic soy agar (TSA; Bacton Dickinson, Franklin Lakes, NJ, USA). A single colony was used to inoculate liquid Luria–Bertani (LB; Bacton Dickinson, Franklin Lakes, NJ, USA) medium at 37 °C to grow the culture until mid-log phase. For the testing, 50 mg of lyophilized *B. globigii* powder (US Army Edgewood Chemical Biological Center, Edgewood, MD, USA) were suspended in 5 mL of Milli-Q water by vigorous vortexing and were centrifuged at 2880× *g* for 7 min to remove traces of the culture medium. The supernatant was removed, and the pellet was resuspended in Milli-Q water with 5% Phosphate Buffer Saline (PBS, pH 7.4) to prepare a stock suspension. Single spore aerosol was generated by the six-jet Collison nebulizer operated at an air pressure of 138 kPa (20 psi gage pressure). For each aerosolization and collection test, a fresh batch (30 mL) of the same type of suspension of about 1 × 10^8^ CFU/L concentration was used. The particle size output from the nebulizer was monitored with the Aerodynamic Particle Sizer (APS; TSI, Burnsville, MN, USA) to ensure that the aerosol was monodisperse and of constant concentration. Microscopy image of an intact spore in the prepared spore suspension can be found in Supplementary Materials Figure S1.

### 2.3. Aerosolization and Bioaerosol Collection

For each trial, 30 mL of fresh suspension was loaded into the nebulizer. Nebulization periods ranged from 5, 10, 15, 30, or 45 min, depending on the experimental conditions. An APS was used to determine the size distribution of the aerosols. The aerosolized bacteria were collected into sterile Milli-Q water by the WWC at 100 L/min airflow rate.

For the high-velocity sampling, bacterial aerosols were collected with the LCP-WWC at 300 L/min airflow. The samples were collected in 0.01% Tween-20 and subjected to 10 min simultaneous aerosolization. Each sample was then divided into two portions; one was analyzed directly and denoted as Tween, while the other was diluted with PBS (pH 7.4) to reach a final concentration of 10% and denoted as Tween + 10PBS.

*B. globigii* suspensions were prepared in Milli-Q water with 5% PBS (osmolality ~14.4 mOsm/L) for standard aerosolization. For LCP-WWC collection, 10% PBS (~29 mOsm/L) was used to alleviate osmotic stress during high-velocity impaction, and 0.01% Tween-20 was included to improve recovery of spores adhering to the cyclone walls. Matched no-aerosol controls (stocks) were analyzed to confirm that observed changes in culturability or antibiotic susceptibility were due to aerosolization stress rather than matrix-induced effects.

### 2.4. Plating and Antibiotic Susceptibility

The collected bioaerosols along with the *B. globigii* stock and nebulized suspension were plated on TSA and incubated overnight at 37 °C before the Colony Forming Units (CFUs) were counted. Antibiotic susceptibility of samples was evaluated using the Kirby–Bauer disk diffusion method (BBL™ Sensi-Disc™ dispenser; Becton Dickinson, Franklin Lakes, NJ, USA) on Mueller–Hinton agar. Each plate was tested against eight commonly used antibiotics, including cell wall synthesis inhibitors: ampicillin (AM, 10 µg), cefoperazone (CFP, 75 µg), cephalothin (CF, 30 µg), and imipenem (IPM, 10 µg); protein synthesis inhibitors: gentamicin (GM, 10 µg) and tetracycline (TE, 30 µg); a DNA synthesis inhibitor: ciprofloxacin (CIP, 5 µg); and a folic acid synthesis inhibitor: sulfamethoxazole–trimethoprim (SXT, 23.75/1.25 µg). The LCP-WWC collected samples were plated repeatedly on Days 0, 2, 5, 10, and 15 to account for dormancy and recovery. A previously established protocol was followed to determine the resistance (R), sensitivity (S), or intermediate (I) response of the samples to specific antibiotics based on the inhibitory area around the disk [37]. The Kirby–Bauer tests were conducted with four replicates. All samples were incubated overnight at 37 °C in LB culture to stationary growth (OD600 = 1.2) and plated on TSA plates. Table S1 in the Supplementary Materials shows the standard raw zone diameters for different strains in the disk diffusion test that was used to evaluate the R/I/S susceptibility.

### 2.5. DNA Extraction and Quantitative Polymerase Chain Reaction

Sample DNA was extracted using a previously described alkaline lysis method [38]. Quantitative polymerase chain reaction (qPCR) assay was performed using 10 µL reaction mixture containing 3 µL of extracted DNA, 1 µL each of 100 µM forward and reverse primers (16S rDNA gene 1369 forward 5′ AAGTCGTAACAAGGT 3′ and 1492 reverse 5′ ACCTTGTTACGACTT 3′, 123 bp fragment) [39], and 5 µL of Power SYBR Green PCR 2x Master Mix (Applied Biosystems, Waltham, CA). Amplification was carried out with an initial denaturation step at 95 °C for 10 min, followed by 40 cycles at 95 °C for 15 s and 60 °C for 60 s, with a final hold at 4 °C. The amplification process was followed by the construction of a dissociation curve (melting curve) for the range 60 °C to 95 °C to detect any nonspecific amplification, including primer–dimer related positive results. The dissociation of the DNA strands during heating was indicated by a large reduction in fluorescence at the specific melting temperature (Tm). The average Tm was 82.4 °C.

Appropriate dilutions of the *B. globigii* vegetative cell and spore stock suspensions were used to generate standard calibration curves for qPCR analysis. The fresh mid-log phase stock suspensions were not subjected to any sampling stress. Based on the LIVE/DEADR BacLight^TM^ Bacterial Viability Kit (Invitrogen Molecular Probes, Carlsbad, CA, USA) staining, the number of culturable cells was directly proportional to the total cell count, with at least 90% of cells viable. Each calibration data point, representing the relationship between cycle threshold (Ct) and cell concentration, was determined from three replicate measurements, and the least squares method was used to generate the best fit line. Standard curves were established using *B. globigii* vegetative cells and spores with known CFU counts to convert to genomic copy numbers (GCNs) after accounting for the presence of two copies of the 16S rDNA region in *B. globigii* genomes [40]. The least squares best fit for the calibration curve is Ct = −1.996ln (cCFU) + 8.8964, where cCFU = CFU/mL. In applying the calibration curve to a sample, a reading Ct was obtained from the qPCR thermocycler, and the corresponding cell concentration was calculated: cCFU = exp((8.8964 − Ct)/1.996). The R^2^ = 0.9742.

### 2.6. Data Analysis and Statistical Significance

The enrichment ratio was calculated to compare bacterial culturability with total genomic abundance. Specifically, it was defined as the ratio between the culture-based counts (CFU/m^3^ of air) and the genomic copy numbers (GCN/m^3^ of air) measured in aerosols relative to those in the fresh culturable stock bacteria aerosolized into the chamber. In other words, the log_10_(CFU/m^3^ of aerosol sample ÷ CFU/m^3^ of stock) was compared to the log_10_(GCN/m^3^ of aerosol sample ÷ GCN/m^3^ of stock) to assess the relative enrichment or loss of culturability during aerosolization and collection. Comparisons between stock samples and collected aerosol samples were also performed to assess changes in culturability and resistance. Statistical significance analysis was carried out with non-parametric one-way analysis of variance (ANOVA) using the Kruskal–Wallis test among multiple groups, and then post hoc pairwise comparisons using Dunn’s multiple-comparison procedure were performed to further delineate which groups were statistically significantly different. For all comparisons, data was considered statistically significant if *p*-value was less than 0.05 (*p* < 0.05).

### 2.7. Computational Fluid Dynamics Modeling

Computational Fluid Dynamics (CFD) modeling was performed following experiments. The input parameters for the modeling were selected based on the air property parameters measured by anemometer (TSI Inc., Burnsville, MN, USA) and HOBOware data loggers (Version 3.7.26). The air velocity was measured at every inch vertically at the inlet, in the center, and outlet of the chamber. The SolidWorks 2023 CAD (Computer-Aided Design) software was used for developing a three-dimensional (3D) model of the chamber, including an inlet, outlet, potential leakage points and the propeller inside the chamber. The 3D model of the chamber with propeller was imported to the ANSYS Workbench (Version 2024 R1) for further processing, including naming of boundaries, mesh creating, and simulations using Fluent. Three meshes of the chamber geometry were created, containing 0.6 million, 1 million, and 3 million tetrahedral cells. A grid independence study was performed using the three generated meshes to compare the velocity profile which indicated minimal differences between the 1 million and 3 million meshes (Supplementary Materials Figure S2). Therefore, further simulations were performed with the mesh containing 1,610,505 cells. The Shear Stress Transport k-ω (SST k-ω) turbulence model was used based on Navier–Stokes equations with a Second Order Upwind, Coupled solver. The second order upwind scheme was used for density, momentum, turbulent kinetic energy, turbulent dissipation rate, and energy. The spatial discretization of pressure was implemented using a second order scheme, which utilizes more information from the neighboring grid cells to calculate pressure gradients, resulting in improved accuracy in capturing pressure variations across the domain. The transport equations for turbulent kinetic energy (k) and specific dissipation rate (ω) are shown in Equations (1) and (2), used by ANSYS Fluent (Version 2024 R1):

(1) $$\frac{𝜕}{𝜕t}\left(\rho k\right)+\frac{𝜕}{𝜕x_{i}}\left(\rho ku_{i}\right)=\frac{𝜕}{𝜕x_{j}}\left(\Gamma_{k}\frac{𝜕k}{𝜕x_{j}}\right)+{\tilde{G}}_{k}-Y_{k}+S_{k}$$

(2) $$\frac{𝜕}{𝜕t}\left(\rho \omega \right)+\frac{𝜕}{𝜕x_{i}}\left(\rho \omega u_{i}\right)=\frac{𝜕}{𝜕x_{j}}\left(\Gamma_{\omega }\frac{𝜕\omega }{𝜕x_{j}}\right)+G_{\omega }-Y_{\omega }+D_{\omega }+S_{\omega }$$

The initial conditions and simulation parameters for the model are listed in Table 1.

## 3. Results

Size distribution of particle injections at the chamber inlet was analyzed using the APS with measurements shown in Table 2 based on aerosolization periods. The average droplet diameter from the 5 min aerosolization period was the highest (0.990 µm) compared to the other aerosolization periods of 10, 15, 30, and 45 min, which were between 0.7 and 0.8 µm. This pattern was different from the previous study that aerosolized the gram-negative *E. coli* under the same conditions, where larger average droplet diameters greater than 1 µm were observed at 10 min and 15 min aerosolization [14].

**Figure 1:**
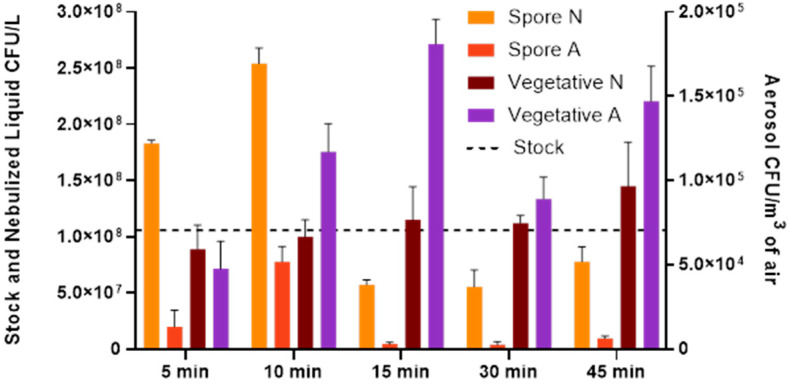
Mean culturable counts (CFU/L) are presented for the *B. globigii* stock suspension (dotted line), the nebulized liquid samples (N), and the aerosol samples collected (A) at CFU/m^3^ for vegetative cells and spores. Tests were performed with four replicates.

**Figure 2:**
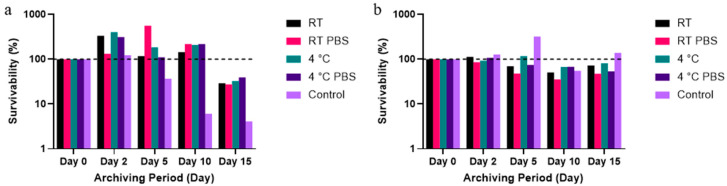
Survivability of *B. globigii* collected using the LCP-WWC with or without the addition of 10% PBS, archived at different temperatures for 15 days. (**a**) Vegetative cell and (**b**) spore survivability expressed as a percentage of the CFU counts from the corresponding Day 0 samples, indicated by the dotted line. RT denotes room temperature of 21 to 23 °C.

The culturability of *B. globigii* spores and vegetative cells was compared after aerosolization for 5, 10, 15, 30, and 45 min with the WWC collection and after 10 min aerosolization with high-pressure and velocity collection using the LCP-WWC (Figure 1 and Figure 2). In Figure 2, the survivability of each sample was determined by normalizing the CFU counts measured at each time point (Day 2, 5, 10, 15) to the Day 0 CFU value of the same sample type. The Day 0 CFU value was set as 100% survival, with values greater than 100% indicating increased CFUs relative to the baseline and values below 100% indicating reduced survival.

The culturable counts of vegetative cells and spores in the nebulized and air samples after various aerosolization time periods were normalized based on the stock and compared. The culturability of aerosolized vegetative cells showed variation but was not significantly different over the five aerosolization times (*p* > 0.05). The higher CFU/m^3^ of air values were vegetative cells aerosolized for 10, 15, and 45 min, while the least culturable vegetative cells were detected in the 5 min and 30 min tests with 4.76 × 10^4^ and 8.19 × 10^4^ CFU/m^3^ of air, respectively. For shorter term aerosolization (5 and 10 min), spores and vegetative cell aerosols behaved similarly, but for the longer aerosolization periods of 15–45 min, the vegetative cells appeared more culturable, at 1.81 × 10^5^, 8.91 × 10^4^, and 1.47 × 10^5^ CFU/m^3^ respectively, while spore culturability was lower, at approximately 3.11 × 10^3^, 2.58 × 10^3^, and 6.30 × 10^3^ CFU/m^3^ of air. The culturability of the spores in the air samples were significantly different among the five different aerosolization times (*p* = 0.0024). Dunn’s multiple comparisons further revealed a significant difference between 10 min and 15 min, and 10 min and 30 min aerosolization, with *p*-values of 0.023 and 0.0042, respectively.

Compared to *E. coli* cells aerosolized under the same operating conditions in our previous study [15] and collected with the LCP-WWC, the culturable counts for *B. globigii* vegetative cells and spores were nearly two orders of magnitude lower than for *E. coli* cells in relation to Day 0 samples (Figure 2a,b). The culturability of vegetative cells stored at room temperature (RT), both with and without a 10% PBS amendment, remained above 100% throughout Day 10, whereas for *E. coli* samples it maintained culturability above 100% for all 15 days. In contrast, spore samples showed culturability above 100% only on Days 2 and 5. This increase early in storage likely reflects germination and outgrowth of a fraction of spores under favorable nutrient conditions, while the remaining spores remained dormant and non-culturable. In the *E. coli* samples stored at 4 °C with no amendments, the percentage of survivability decreased by a magnitude in a period of 10 and 15 days [15].

*B. globigii* spore survivability stayed more consistent than for vegetative cells. By Day 15, the survivability for vegetative cells was 50% of the RT spores and of all vegetative cells on Day 15. Due to the spores’ ability to survive harsh environments, the maintenance of survivability is consistent with their resistance to environmental changes, while vegetative cells were able to grow at most to 562% on Day 5, and then decreased gradually by Day 15 to 50% of the original survivability. There was a significant difference between vegetative cell counts in the Day 0 and Day 15 RT samples.

**Figure 3:**
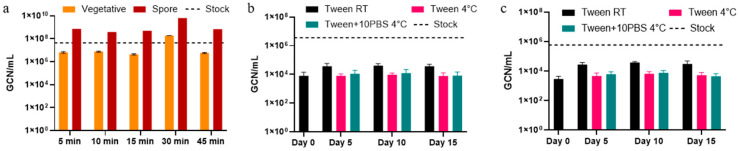
GCN of the *B. globigii* samples from qPCR analysis. (**a**) Aerosols collected using the WWC after five different aerosolization durations. (**b**) Vegetative cells and (**c**) spores archived for 15 days at room temperature (RT) or at 4 °C in Tween, with or without the addition of PBS. The solid line indicates the non-aerosolized stock.

The qPCR results are presented in GCN per milliliter (GCN/mL). Figure 3a shows that the aerosolized cells in the airtight chamber grew approximately four magnitudes higher than the cells aerosolized and collected with the LCP-WWC. Vegetative cells had on average lower GCN/mL values by one magnitude compared to spores. This contrasts with culturability of the spores and vegetative cells, which showed higher CFU/m^3^ of air values in vegetative cells than in spores. This shows that while the presence of spores was greater, the culturability was lower. This pattern suggests that while vegetative cells remained more culturable after aerosolization, they were also more susceptible to DNA damage or loss, resulting in lower GCN recovery. In contrast, spores, though less culturable, likely retained more intact genomic material due to their higher structural resilience to aerosolization stress. In the LCP-WWC testing, the vegetative cell GCN/mL and spore GCN/mL values were nearly identical (Figure 3b,c).

**Figure 4:**
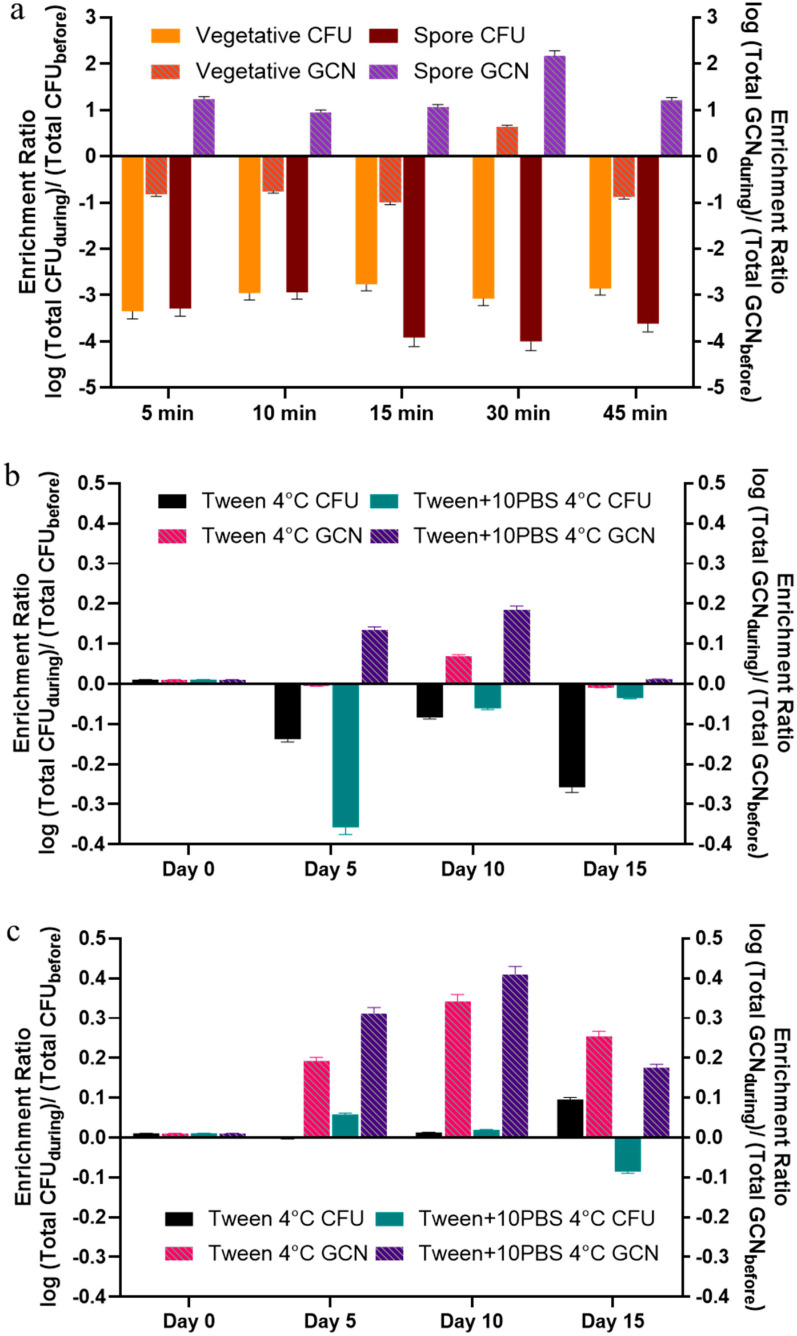
Enrichment ratio of the collected aerosols based on CFUs (left axis) and GCNs (right axis) compared to the stock (**a**) nebulized into the chamber for different time periods, (**b**) vegetative cells, and (**c**) spores collected with the LCP-WWC.

Figure 4 shows the enrichment ratio of total CFUs and GCNs. Spore and vegetative cell enrichment ratios were consistent for most chamber tests, with score ratios becoming more negative in longer aerosolization times. The enrichment ratio reflects the survivability of *B. globigii* and shows that the GCN values reflect a small increase in numbers over time in overall samples but not in culturability, indicating some, but not significant growth in the samples (Figure 4a). There was a significant difference between RT samples on Days 5–15 compared to all other samples (Figure 4b,c). There were approximately three magnitudes less of cell counts in the WWC studies. This is expected, as it indicates that cells were able to utilize time to cope with stress. There was a greater change in the total GCN ratios in the WWC test samples (Figure 4a) compared to the LCP-WWC test samples (Figure 4b,c). While there was a greater change, the GCN values at each time duration were not significantly different from that of the stock.

**Figure 5:**
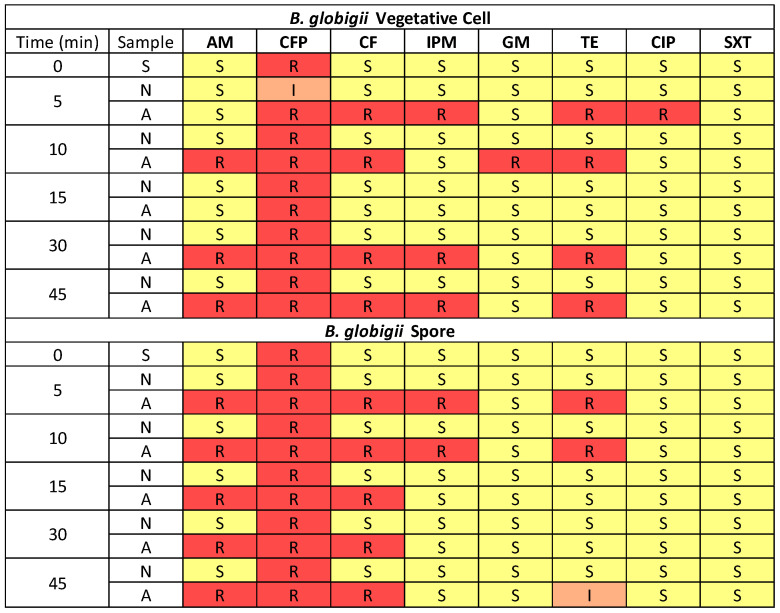
Heat map showing antibiotic susceptibility of *B. globigii* samples aerosolized for 5, 10, 15, 30, and 45 min when collected by WWC. Resistance levels are indicated as Resistant (red), Intermediate (orange), and Susceptible (yellow), based on the Kirby–Bauer test with eight antibiotics. Sample types include stock suspension (S), nebulized liquid (N), and collected aerosol (A).

The antibiotic susceptibility profile was different in *B. globigii* spores and vegetative cells, as shown in Figure 5. The exact inhibition zone diameter of samples can be found in Supplementary Materials Figure S3. Vegetative cells had a broader range of susceptibility in the profile when compared to *E. coli* cells, showing resistance in some cases to every antibiotic except SXT. Spores did not show any resistance to GM, CIP, or SXT. Vegetative cells behaved similarly to the gram-negative *E. coli*, in which there was a period of more favorable conditions and less resistance during 15 min aerosolization. In *E. coli* cells, that range was from 10 to 15 min, but in *B. globigii* cells it was a shorter range of 15 min with resistance increasing again during the 30 and 45 min tests. There was the same proportion of resistance in most samples (5, 10 min) and then at the 15 min period, spores exhibited more resistance. However, for the longer time period of 30 min, vegetative cells maintained more resistance.

**Figure 6:**
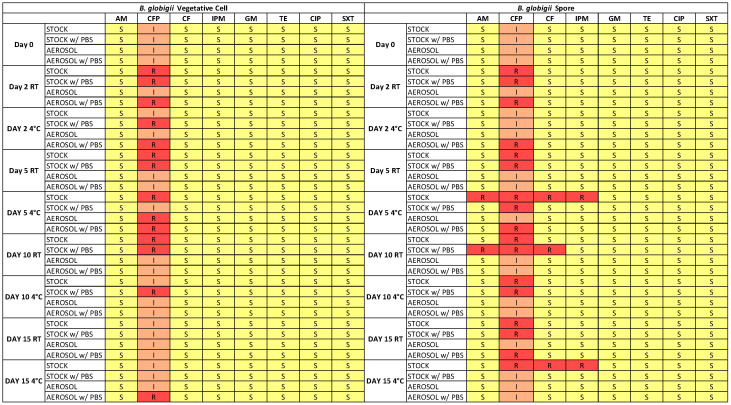
Heat map showing antibiotic susceptibility of *B. globigii* vegetative cells and spores collected with the LCP-WWC and stock suspensions, archived for 15 days at room temperature or 4 °C, with or without PBS. Resistance levels are indicated as Resistant (red), Intermediate (orange), and Susceptible (yellow), based on the Kirby–Bauer test with eight antibiotics.

**Figure 7:**
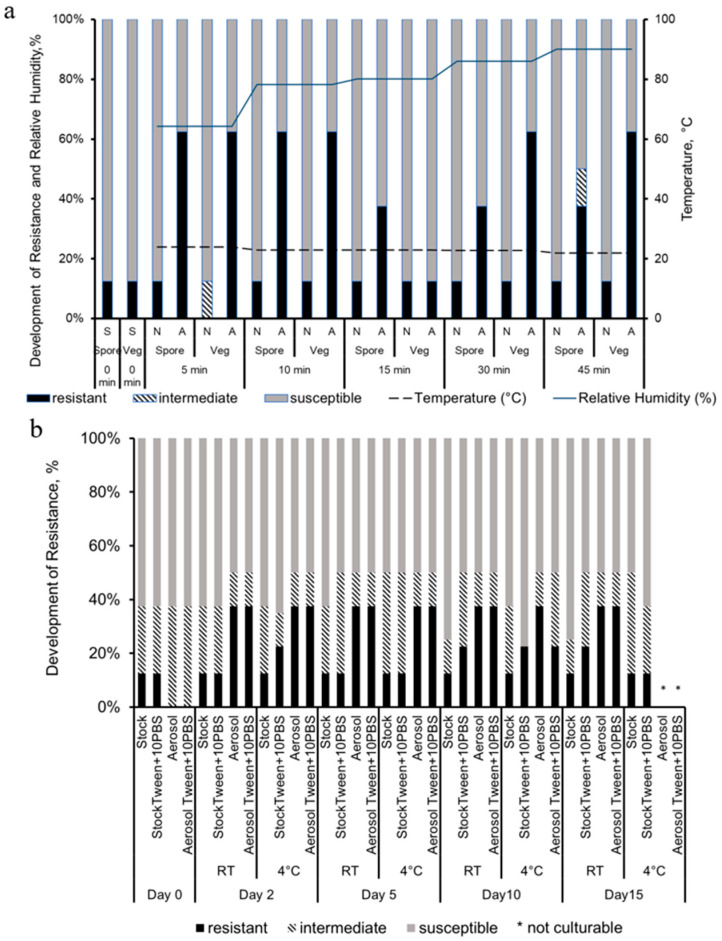

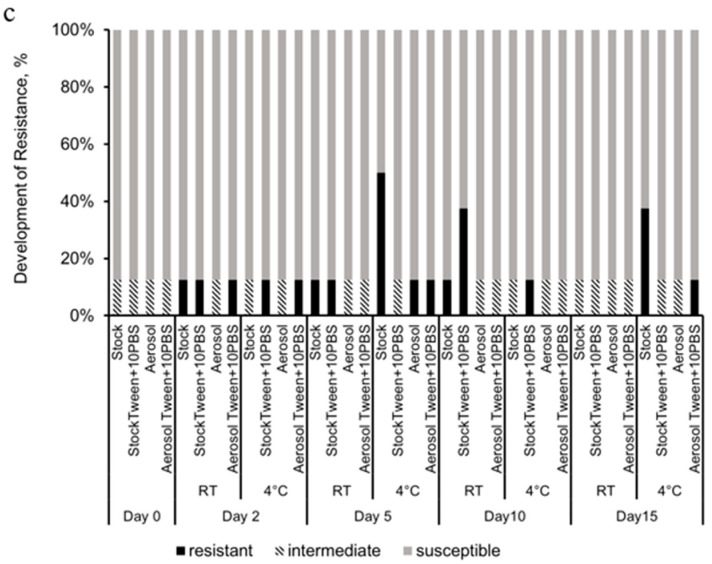
The percentage of antibiotic susceptibility in (**a**) *B. globigii* vegetative cells and spore stock suspensions (S), nebulized liquids (N), and aerosols (A) after 5, 10, 15, 30, and 45 min aerosolization and collection by the WWC, along with the RH (left axis) and temperature (right axis) inside the chamber, during the tests. The percentage of antibiotic susceptibility in (**b**) vegetative cell and (**c**) spore samples collected with the LCP-WWC and archived for 15 days at room temperature or 4 °C, with or without PBS.

Figure 6 shows the antibiotic susceptibility of the *B. globigii* that was collected with the LCP-WWC and archived for 15 days at different conditions. The exact inhibition zone diameter of samples can be found in Supplementary Materials Figure S4. While no strong resistance was detected in the aerosol samples (Figure 7a), some resistance was found in the spore samples after LCP-WWC sampling (Figure 7b,c). This is likely due to the spores’ ability to resist unfavorable environments, and after stress of storage for 5 days, they developed some resistance. The aerosols seemingly were overstressed and not able to evade the antibiotics.

**Figure 8:**
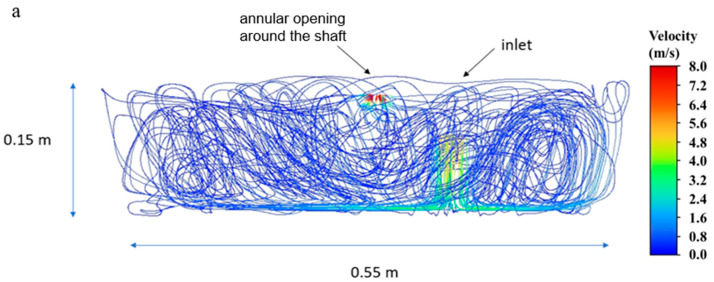

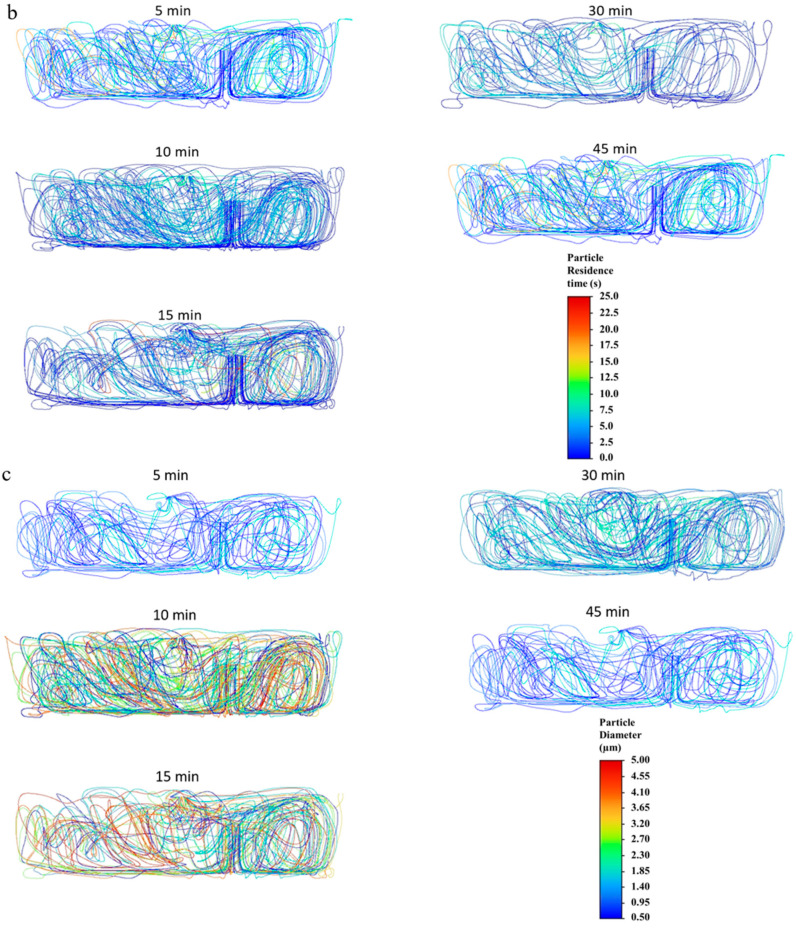
Changes in (**a**) air velocity streamlines, (**b**) particle residence time, and (**c**) particle diameter at 5, 10, 15, 30, and 45 min of the steady state simulations in the environmental chamber.

ANSYS Fluent (Version 2024 R1) simulations were performed following the environmental chamber operating conditions during the experiments. Figure 8 shows the velocity, residence time and diameter of particles. The velocity magnitude was found to be at 0.7 m/s, with the highest velocity localized at the stirring rod (location of steering propeller is indicated as “annular opening around the shaft”) (Figure 8a). The residence time of the aerosolized droplets was between 5 and 45 min (Figure 8b). The droplet diameters ranged in size, from 0.534 µm to 7.2 µm (Figure 8c). With mean diameters based upon Table 2, Figure 8b shows the distribution of the particles’ residence time within the chamber utilized for aerosol testing. Simulation results were validated using APS data [14] with larger particles at 10 and 15 min tests with 1.70 and 1.07 µm diameters, respectively, and relatively similar droplets at 5, 30, and 45 min tests with 0.89, 0.9, and 0.89 µm diameters, respectively (Figure 8c).

## 4. Discussion

Spore-forming bacteria are known to be more resistant, metabolically inactive, and able to withstand harsh environments. This study showed when comparing the culturability of *B. globigii* spores and vegetative cells to *E. coli* cells, there are optimal times of aerosolization that produce less antibiotic-resistant bacteria. Very short or long-term durations of aerosolization create more stress on the bacteria, either due to the lack of sufficient time to activate stress responses during brief exposure or because the cells are not able to maintain coping strategies for prolonged aerosolization. While the current results were obtained using a surrogate organism in a controlled chamber system, the observed trends suggest that timely air exchange, such as the CDC recommended 99.9% removal of airborne contaminants within 30 min [41], may be an effective mitigation strategy under real-world conditions. However, this interpretation should be considered a hypothesis-generating method, rather than validation, as particle dynamics and environmental factors differ between controlled systems and actual ventilation settings.

In *B. globigii* spores, there was less culturability but more consistent development of resistance. Samples aerosolized for 5 and 10 min developed resistance to ampicillin, cephalothin, tetracycline, and imipenem. More resistance was developed in vegetative cells, especially for the 5, 30, and 45 min tests. Fifteen minutes was the optimal time for culturability and for resistance, in which there was no developed resistance that was not already in the stock samples, indicating that *B. globigii* spores and vegetative cells were the least stressed. According to our previous study, 10 and 15 min aerosolization samples contained the largest average droplets and most likely allowed a hydro-shell to form around the bacteria cells, protecting them from the harsh environmental stressors [14]. Samples aerosolized for 5 and 10 min collectively were resistant to all the antibiotics, except for sulfamethoxazole–trimethoprim. Vegetative cells aerosolized for 30 and 45 min behaved similarly to spores and developed resistance to ampicillin, cephalothin, tetracycline, and imipenem. Resistance was developed to all cell wall synthesis inhibitors throughout spore and vegetative tests continuing to validate our claims that cells enact the first line of defense when introduced to aerosolization stress [14]. The resistance in samples that were less culturable but still showed resistance was always toward cell wall synthesis inhibitors in the long-term aerosolization tests. Least culturable vegetative cells were found after 5 and 30 min, but they showed resistance to a wider range of antibiotics including DNA synthesis and cell wall synthesis inhibitors.

Consideration must be given to the culturability differences, as vegetative cells would be more culturable than spores, which are metabolically inactive and may have exhibited stunted growth once finding favorable growth environments on the TSA plates and the incubator. While culturability was expected to be affected, it was still clear that aerosolization for 15 min or longer was harder on spores, where the shorter 10 min period yielded the highest culturability. The number of culturable counts is important, as the estimated median lethal dose (LD50) of *B. anthracis* ranges from 10 or fewer spores for cutaneous anthrax to 2500–55,000 spores for inhalation anthrax [42,43]. The lack of trends in culturability over different aerosolization durations, where CFU was higher at 10, 15, and 45 min but lower at 30 min, suggests a dynamic pattern between stress exposure and recovery potential. This is similar to the results of the previous study with *E. coli* that showed lower culturability, with the 5 and 30 min tests being noted as critical times during aerosolization [14]. During short aerosolization periods, bacteria may experience initial desiccation or shear stress, yet retain sufficient physiological capacity for repair upon collection. Intermediate duration of 30 min may represent a point of maximal stress where cells cannot fully recover, leading to decreased culturability, whereas extended duration of 45 min allows selection for more resilient subpopulations or induction of stress-response pathways that temporarily enhance survival. The phenomena for adaptive tolerance under environmental stresses have been reported for *Bacillus subtilis*, where researchers found that the cells can enter the viable but non-culturable state upon sudden osmotic changes [44].

The LCP-WWC samples showed a larger difference over time in terms of survivability over the 15 day period. Vegetative cells samples consistently maintained the same magnitude for Days 2, 5, and 10, except for the control samples. However, Day 15 exhibited a decrease nearly by a magnitude. Spore viability stayed consistent from Day 0 to Day 2, with no significant decrease in culturability for Days 5–15. This is different from *E. coli* cells, where an increase of two magnitudes was detected for Days 2, 5, and 10 for most samples, followed by a decrease to only being one magnitude greater than the Day 0 samples on Day 15.

Vegetative cells only developed more resistance to cefoperazone. On Days 2–15, stock and aerosol samples of vegetative cells show full resistance to cefoperazone, as opposed to intermediate resistance on Day 0. Less resistance was found on Day 15 compared to Days 2–10, indicating that these samples were more stressed and unable to maintain their resistance. Spores showed resistance in stock and aerosol samples similarly to vegetative cells to cefoperazone in Days 2–15. There was also development of resistance in stock samples on Days 5–15 that could indicate that the storage of *B. globigii* spores over longer periods of time induced some stressors in which culturability was maintained but resistance/susceptibility was not. This is very different from *E. coli* cells which showed resistance in aerosols for Days 2–10 to ampicillin and cephalothin, and started with resistance in stock samples to cefoperazone.

As opposed to culturability, which only shows a fraction of cells present, the GCN values were also calculated and showed that there was an increase and then plateau in GCN/mL by Day 10. Ratios showed more recovery of GCN than CFU, which is expected. Although we expected to see more resistance in spores, more resistance was detected in vegetative cells aerosolized for different durations. Spores’ robust structural layers and DNA protection mechanisms allow them to better preserve genomic material than vegetative cells under physical and oxidative stress [45,46]. The higher GCN observed for spores compared to vegetative cells in some conditions likely reflect their resistance to DNA degradation during aerosolization and subsequent storage. LCP-WWC testing showed that more spores became resistant due to stress, however, not from aerosolization. Although spores are metabolically dormant and do not actively acquire new genetic mutations during aerosolization, they can still exhibit apparent resistance or tolerance once germinated. This phenomenon likely reflects phenotypic adaptation, rather than genetic resistance. Aerosolization and desiccation stress can induce structural or physiological alterations in the spore coat and cortex, leading to delayed germination and transient tolerance to antibiotics after revival [47,48]. The increased antibiotic tolerance observed in spores in the current study likely arises from stress-induced physiological adaptations, rather than resistance acquisition. Future studies should aim to investigate this hypothesis by monitoring spore germination dynamics and assessing whether tolerance patterns change during outgrowth.

## Figures and Tables

**Table 1:** CFD initial conditions and simulation parameters.

Parameter	Value
Temperature	27 °C
Inlet	Velocity Inlet (4.937 m/s)
Natural Leakage Outlet	Pressure Outlet (0 Pa)
Walls	Stationary, No Slip
Tolerance (x)	10^−3^
Tolerance (y)	10^−3^
Tolerance (P)	10^−3^
Tolerance (velocity)	10^−3^
Tolerance (k)	10^−3^
Tolerance (ω)	10^−3^

**Table 2:** Droplet characteristics during 5, 10, 15, 30, and 45 min aerosolization periods.

Aerosolization Time, min	Mass Median Diameter, µm	Average Diameter, µm	Particle Concentration, Number/cm^3^
5	0.936	0.990	1498
10	0.630	0.753	3026
15	0.643	0.785	1232
30	0.656	0.721	664
45	0.633	0.705	1436

## Data Availability

The original contributions presented in this study are included in the article/Supplementary Material. Further inquiries can be directed to the corresponding author.

## Supplementary Materials

The following supporting information can be downloaded at: https://www.mdpi.com/article/10.3390/microorganisms13112532/s1, Figure S1: Cryo-TEM image of an intact *Bacillus globigii* cell with spore; Figure S2: Velocity in the y direction of the center line in the inlet of the chamber compared for a coarse (0.6 million cells), medium (1 million cells) and fine (3 million cells) meshes; Figure S3: Inhibition zone diameter (mm) of *B. globigii* samples aerosolized for 5, 10, 15, 30, and 45 min from the Kirby-Bauer test with eight antibiotics. Sample types include stock suspension (S), nebulized liquid (N), and collected aerosol (A). Numbers shown are average of at least three replicates; Figure S4: Inhibition zone diameter (mm) of *B. globigii* samples collected with the LCP-WWC and stock suspensions, archived for 15 days at room temperature or 4 °C with or without PBS. Numbers shown are average of at least three replicates; Table S1: Antimicrobials used in the antibiotic disc dispenser and the R/I/S zone diameters.
