# Supplementary material for: Aerosolization Affects Bacillus globigii Vegetative Cell and Spore Behaviors
Source: Microorganisms. 2025 Nov 5;13(11):2532. doi: 10.3390/microorganisms13112532 (PMC12654006; doi:10.3390/microorganisms13112532)
Supplement: Supplementary file 1 [file microorganisms-13-02532-s001.zip › microorganisms-3883730-supplementary.pdf]

Supplementary Materials

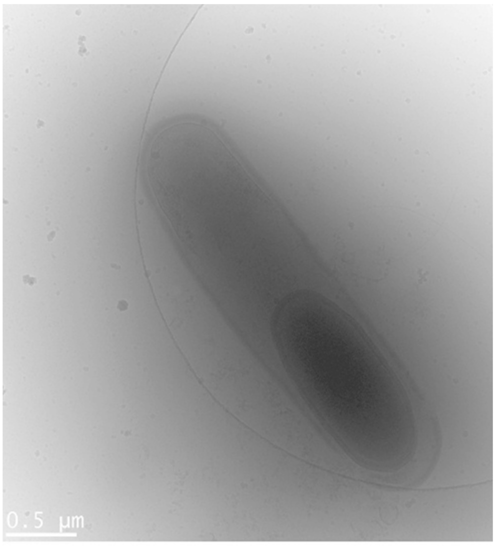

Figure S1. Cryo-TEM image of an intact *Bacillus globigii* cell with spore.

Table S1. Antimicrobials used in the antibiotic disk dispenser and their R/I/S zone diameters.

Antibiotic (Antimicrobial Agent)	DISK CODE	Resistant (R) (< or = mm)	Intermediate (I) (mm)	Susceptible (S) (= or > mm)
Ampicillin (other)	AM-10	11	12-13	14
Cefoperazone	CFP-75	27	28-34	35
Cephalothin	CF-30	14	15-17	18
Ciprofloxacin	CIP-5	15	16-20	21
Gentamycin	GM	12	13-14	15
Imipenem	IPM	13	14-15	16
Sulfamethoxazole-trimethoprim	SXT	10	11-15	16
Tetracycline	Te-30	14	15-18	19

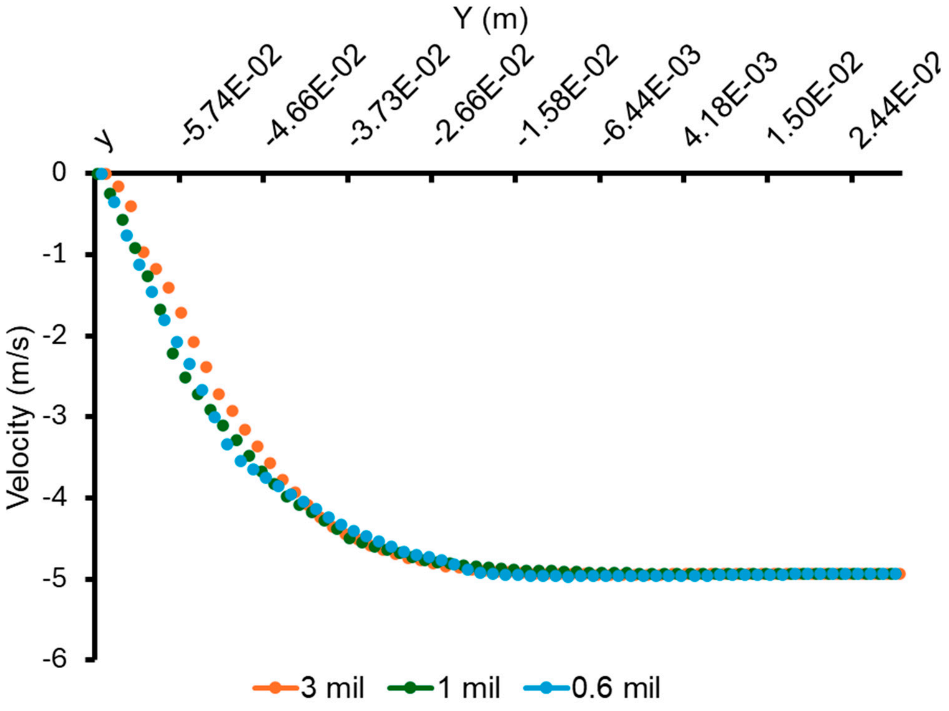

Figure S2. Comparing velocity in the y direction from the center line at the inlet of the chamber for coarse (0.6 million cells), medium (1 million cells), and fine (3 million cells) meshes.

Time (min)	Sample	AM	CFP	CF	IPM	GM	TE	CIP	SXT
		***B. globigii* Vegetative Cell**							
0	S	19.2	25.2	24.8	33.2	29.0	22.0	29.6	24.8
5	N	15.3	33.5	27.5	36.0	30.0	22.5	36.0	26.8
	A	14.5	24.0	0.0	0.0	24.3	4.8	0.0	21.0
10	N	17.8	25.0	22.0	24.3	25.3	26.0	26.0	26.0
	A	0.0	25.3	0.0	34.8	12.5	14.5	29.5	29.0
15	N	22.0	25.3	31.8	35.0	23.8	24.0	25.7	28.0
	A	22.5	25.8	39.5	35.5	24.5	23.0	25.3	28.0
30	N	20.0	22.5	27.5	31.5	25.0	22.5	28.0	26.8
	A	3.5	18.5	0.0	0.0	24.0	4.8	22.8	21.0
45	N	20.0	22.5	27.5	31.5	25.0	22.5	28.0	26.8
	A	3.5	18.5	0.0	0.0	24.0	4.8	22.8	21.0
		***B. globigii* Spore**							
0	S	18.0	24.7	30.7	25.3	30.7	38.0	26.7	30.3
5	N	20.7	26.7	35.3	27.0	32.0	40.0	24.5	32.0
	A	0.0	25.0	0.0	9.2	26.3	9.3	24.0	16.0
10	N	19.3	26.0	34.7	27.0	30.3	39.3	24.0	32.3
	A	0.0	23.7	0.0	11.7	26.7	6.0	25.3	17.3
15	N	19.3	26.7	34.0	24.7	32.3	36.7	27.3	29.7
	A	7.3	24.7	0.0	21.3	28.7	29.7	22.0	23.7
30	N	17.7	25.3	34.0	25.0	30.7	36.7	26.3	29.0
	A	8.7	24.7	0.0	22.3	29.3	35.3	23.7	27.3
45	N	19.7	27.3	36.0	25.7	29.3	37.3	26.7	28.0
	A	0.0	22.7	0.0	22.0	25.7	17.0	23.8	27.0

Figure S3. Inhibition zone diameter (mm) of *B. globigii* samples aerosolized for 5, 10, 15, 30, and 45 min from the Kirby-Bauer test with eight antibiotics. Sample types include stock suspension (S), nebulized liquid (N), and collected aerosol (A). Numbers shown are average of at least three replicates.

B. globigii Vegetative Cell									
		AM	CFP	CF	IPM	GM	TE	CIP	SXT
Day 0	STOCK	22.0	30.0	36.0	39.0	26.0	26.0	30.0	26.0
	STOCK w/ PBS	22.0	30.0	36.0	39.0	26.0	26.0	30.0	26.0
	AEROSOL	22.0	30.0	40.0	36.0	25.3	24.0	25.3	26.0
	AEROSOL w/ PBS	22.0	30.0	40.0	36.0	25.3	24.0	25.3	26.0
Day 2 RT	STOCK	19.0	24.0	36.0	38.0	30.0	24.0	32.0	26.0
	STOCK w/ PBS	22.0	24.0	39.0	36.0	24.0	25.0	28.0	26.0
	AEROSOL	21.3	28.0	40.7	37.0	24.7	24.3	26.7	28.7
	AEROSOL w/ PBS	21.7	27.7	40.7	36.0	25.3	24.3	26.7	29.7
DAY 2 4°C	STOCK	22.0	28.0	41.0	38.0	26.0	28.0	26.0	28.0
	STOCK w/ PBS	22.0	25.0	39.0	36.0	26.0	22.0	27.0	30.0
	AEROSOL	20.7	28.0	42.0	36.0	25.7	23.0	26.3	26.0
	AEROSOL w/ PBS	21.0	26.0	40.7	37.7	24.3	23.3	26.7	28.0
Day 5 RT	STOCK	16.0	17.0	22.0	37.0	28.0	22.0	30.0	30.0
	STOCK w/ PBS	15.0	21.0	26.0	42.0	28.0	27.0	30.0	33.0
	AEROSOL	20.0	28.7	40.0	35.3	25.3	24.0	25.3	26.7
	AEROSOL w/ PBS	22.0	31.3	41.7	37.3	26.7	25.3	30.0	29.3
DAY 5 4°C	STOCK	16.0	26.0	40.0	36.0	24.0	24.0	26.0	28.0
	STOCK w/ PBS	21.0	32.0	42.0	40.0	25.0	26.0	28.0	30.0
	AEROSOL	15.0	23.3	36.7	37.3	23.3	24.0	25.7	28.0
	AEROSOL w/ PBS	12.7	27.0	27.0	27.0	27.3	25.3	38.0	37.3
DAY 10 RT	STOCK	19.0	20.0	24.0	41.0	28.0	25.0	30.0	32.0
	STOCK w/ PBS	18.0	22.0	22.0	40.0	28.0	24.0	30.0	30.0
	AEROSOL	15.3	28.0	40.0	38.0	25.3	25.7	26.7	27.3
	AEROSOL w/ PBS	15.3	28.0	40.0	38.0	25.3	25.7	26.7	27.3
DAY 10 4°C	STOCK	23.0	30.0	44.0	38.0	24.0	22.0	26.0	28.0
	STOCK w/ PBS	17.0	26.0	34.0	34.0	26.0	26.0	30.0	27.0
	AEROSOL	22.3	32.0	42.0	38.7	26.7	25.7	26.7	29.3
	AEROSOL w/ PBS	11.3	30.0	30.0	25.0	26.0	23.0	42.0	37.0
DAY 15 RT	STOCK	16.0	30.0	43.0	42.0	30.0	27.0	30.0	32.0
	STOCK w/ PBS	16.0	20.0	18.0	40.0	32.0	24.0	30.0	32.0
	AEROSOL	24.7	33.3	44.3	39.3	28.0	27.0	29.3	30.0
	AEROSOL w/ PBS	23.3	31.0	41.7	39.3	25.7	25.3	32.0	29.0
DAY 15 4°C	STOCK	24.0	32.0	43.0	40.0	28.0	31.0	28.0	32.0
	STOCK w/ PBS	24.0	32.0	44.0	40.0	26.0	26.0	30.0	30.0
	AEROSOL	21.0	30.0	42.0	40.0	26.3	26.7	29.3	30.7
	AEROSOL w/ PBS	23.0	23.0	30.7	26.0	29.3	25.7	43.0	30.0

B. globigii Spore									
		AM	CFP	CF	IPM	GM	TE	CIP	SXT
Day 0	STOCK	24.0	30.0	39.0	24.0	28.0	36.0	26.0	26.0
	STOCK w/ PBS	24.0	30.0	39.0	24.0	28.0	36.0	26.0	26.0
	AEROSOL	20.6	29.3	38.6	24.0	27.3	37.0	25.3	25.6
	AEROSOL w/ PBS	20.6	29.3	38.6	24.0	27.3	37.0	25.3	25.6
Day 2 RT	STOCK	16.0	24.0	27.0	20.0	26.0	38.0	26.0	28.0
	STOCK w/ PBS	22.0	24.0	27.0	22.5	26.0	24.0	24.0	23.0
	AEROSOL	21.0	29.3	40.7	23.3	26.0	36.0	24.7	28.7
	AEROSOL w/ PBS	20.3	27.3	40.7	25.3	27.0	36.0	26.0	25.3
DAY 2 4°C	STOCK	21.0	34.0	40.0	24.0	26.0	36.0	24.0	26.0
	STOCK w/ PBS	23.0	30.0	40.0	19.5	26.0	37.0	24.0	26.0
	AEROSOL	20.3	28.0	40.7	23.7	26.7	36.3	24.0	26.0
	AEROSOL w/ PBS	21.0	24.3	41.0	22.3	25.7	35.3	26.3	28.0
Day 5 RT	STOCK	23.0	21.0	26.0	26.0	32.0	40.0	30.0	30.0
	STOCK w/ PBS	16.0	19.0	22.0	26.0	26.0	40.0	28.0	32.0
	AEROSOL	19.0	29.3	42.0	24.3	26.0	34.0	24.7	28.7
	AEROSOL w/ PBS	16.3	29.3	40.0	22.0	25.0	35.3	23.7	28.0
DAY 5 4°C	STOCK	0.0	21.0	11.0	13.0	32.0	24.0	25.0	31.0
	STOCK w/ PBS	15.0	26.0	38.0	24.0	27.0	36.0	28.0	26.0
	AEROSOL	21.3	30.0	40.0	25.0	27.3	39.0	26.0	28.0
	AEROSOL w/ PBS	21.3	19.7	40.7	23.7	26.0	36.7	24.7	29.0
DAY 10 RT	STOCK	17.0	19.0	24.0	26.0	30.0	28.0	25.0	26.0
	STOCK w/ PBS	0.0	20.0	14.0	24.0	28.0	40.0	30.0	32.0
	AEROSOL	20.7	29.7	42.0	24.3	26.0	37.7	25.3	28.7
	AEROSOL w/ PBS	18.7	30.0	40.0	24.0	25.3	39.0	25.3	28.7
DAY 10 4°C	STOCK	20.0	20.0	22.0	26.0	30.0	42.0	30.0	32.0
	STOCK w/ PBS	15.0	20.0	22.0	26.0	30.0	40.0	30.0	32.0
	AEROSOL	21.2	30.0	41.3	26.0	26.0	38.7	26.0	30.0
	AEROSOL w/ PBS	17.8	28.0	42.7	24.7	27.3	37.3	26.0	31.7
DAY 15 RT	STOCK	15.0	20.0	24.0	24.0	30.0	23.0	26.0	24.0
	STOCK w/ PBS	16.0	18.0	24.0	24.0	30.0	36.0	32.0	24.0
	AEROSOL	24.0	30.0	43.0	25.3	28.0	39.3	25.7	29.7
	AEROSOL w/ PBS	21.5	23.7	31.3	25.3	30.7	34.0	28.7	23.7
DAY 15 4°C	STOCK	26.0	20.0	10.0	13.0	32.0	40.0	28.0	32.0
	STOCK w/ PBS	22.0	32.0	44.0	26.0	30.0	40.0	26.0	30.0
	AEROSOL	21.3	33.3	44.0	22.7	26.0	40.7	24.0	30.7
	AEROSOL w/ PBS	20.8	28.7	40.7	23.3	32.0	39.0	25.7	27.0

Figure S4. Inhibition zone diameter (mm) of *B. globigii* samples collected with the LCP-WWC and stock suspensions, archived for 15 days at room temperature or 4 °C, with or without PBS. Numbers shown are average of at least three replicates.
